# Autophagy Regulatory Genes MET and RIPK2 Play a Prognostic Role in Pancreatic Ductal Adenocarcinoma: A Bioinformatic Analysis Based on GEO and TCGA

**DOI:** 10.1155/2020/8537381

**Published:** 2020-11-05

**Authors:** Xingyu Li, Zhiqiang Li, Hongwei Zhu, Xiao Yu

**Affiliations:** Department of Hepatobiliary and Pancreatic Surgery II, The Third Xiangya Hospital, Central South University, Changsha City 410013, Hunan Province, China

## Abstract

Pancreatic ductal adenocarcinoma is a common malignant tumor with a poor prognosis. Autophagy activity changes in both cancer cells and microenvironment and affects the progression of pancreatic ductal adenocarcinoma. The purpose of this study was to predict the prognostic autophagy regulatory genes and their role in the regulation of autophagy in pancreatic ductal adenocarcinoma. We draw conclusions based on gene expression data from different platforms: GSE62165 and GSE85916 from the array platform, TCGA from the bulk RNA-seq platform, and GSE111672 from the single-cell RNA-seq platform. At first, we detected differentially expressed genes in pancreatic ductal adenocarcinoma compared with normal pancreatic tissue based on GSE62165. Then, we screened prognostic genes based on GSE85916 and TCGA. Furthermore, we constructed a risk signature composed of the prognostic differentially expressed genes. Finally, we predicted the probable role of these genes in regulating autophagy and the types of cell expressing these genes. According to our screening criteria, there were only two genes: MET and RIPK2, selected into the development of the risk signature. However, evaluated by log-rank tests, receiver operating characteristic curves, and calibration curves, the risk signature was worth considering its clinical application because of good sensitivity, specificity, and stability. Besides, we predicted that both MET and RIPK2 promote autophagy in pancreatic ductal adenocarcinoma by gene set enrichment analysis. Analysis of single-cell RNA-seq data from GSE111672 revealed that both MET and RIPK2 were expressed in cancer cells while RIPK2 was also expressed in monocytes and neutrophils. After comprehensive analysis, we found that both MET and RIPK2 are related to the prognosis of pancreatic ductal adenocarcinoma and provided some associated clues for clinical application and basic experiment research.

## 1. Introduction

Pancreatic cancer has become one of the leading causes of cancer mortality worldwide [1]. Pancreatic ductal adenocarcinoma (PDAC) is the major type of malignant pancreatic neoplasms (more than 80%) [2], with a five-year survival rate at about 5% and median survival time less than one year [3, 4]. Poor prognosis results from loss of surgical opportunities due to the advanced tumor stage at diagnosis and high resistance to chemotherapy and radiation [5, 6]. Although clinicopathological characteristics and some biomarkers have been used to predict patients' prognosis, identification of transcriptional markers and development of novel methodology for accurate prediction of PDAC outcome are still encouraged.

Autophagy plays an important role in both the maintenance of homeostasis and the progress of tumors. In a normal organizational environment, autophagy maintains cell homeostasis to prevent diseases by supporting mammalian development, regulating metabolism at different nutritious states, and disposing damaged proteins and organelles [7]. The effect of autophagy on tumor varies with the stage of tumor. Autophagy suppresses tumor at the initial stage by being involved in removing dysfunctional mitochondria and redox-active aggregates of ubiquitinated proteins, disposal of micronuclei, and degradation of retrotransposing RNAs and responses to genotoxic stress [8], suggesting probable utilization of autophagy inducers as tumor preventive agents. However, in an established tumor, autophagy promotes tumor progression by allowing cancer cells to survive under metabolic stress, supporting metabolic rearrangements, and maintaining cancer stem cells [9]. Both gene products and exogenous substances participate in the regulation of autophagy in tumors, proposing related therapeutic strategies [10, 11].

The effect of autophagy on PDAC is so complicated that both autophagy inducers and autophagy inhibitors have the potential to be used as drugs to treat PDAC [12]. In addition to the influence on tumor cells, autophagy also affects PDAC progression by changing microenvironment [13, 14]. However, there is still a lack of systematic analysis of the prognostic value of autophagy regulatory genes in PDAC and their regulatory effect on autophagy. In this study, we sifted out two autophagy regulatory genes, MET and RIPK2, whose high RNA expressions are both significantly related to poor survival of PDAC patients. Then, we developed a two-gene risk signature based on these two genes. Furthermore, we predicted that both MET and RIPK2 promote autophagy in PDAC and constructed a coexpression network. Finally, we identified the types of cell expressing these genes and proved the upregulation of MET and RIPK2 expression using surgical specimens, providing clues for basic research and potential targets for clinical treatment.

## 2. Materials and Methods

### 2.1. Patient Samples

The gene expression profiles by the array of GSE62165 [15] and GSE85916 and associated clinical data were downloaded from Gene Expression Omnibus (GEO, https://www.ncbi.nlm.nih.gov/geo/). The gene expression RNA-seq-batch effects normalized mRNA data of the Cancer Genome Atlas (TCGA) was downloaded from the University of California Santa Cruz (UCSC) Xena website (https://xenabrowser.net/datapages/) [16]. Gene expression data of 150 PDAC samples was extracted according to the Cancer Genome Atlas Research Network [17, 18]. The associated survival and phenotype data of pancreatic cancer were also downloaded from the UCSC Xena website. The data of single-cell RNA-seq (scRNA-seq) data of GSE111672 [19] was also downloaded from GEO.

### 2.2. Gene Lists

The list of 319 autophagy regulatory genes was obtained from gene set: GO_REGULATION_OF_AUTOPHAGY (M10281) in the Molecular Signatures Database (MSigDB, http://software.broadinstitute.org/gsea/msigdb/index.jsp). The other five gene set files, hallmark gene sets, and Kyoto Encyclopedia of Genes and Genomes (KEGG) gene sets for enrichment analysis were also obtained from MSigDB. The list of 232 human genes and proteins involved in autophagy was obtained from the Human Autophagy Database (HADb, http://www.autophagy.lu/) [20].

### 2.3. Prognostic Analysis

**Figure 1:**
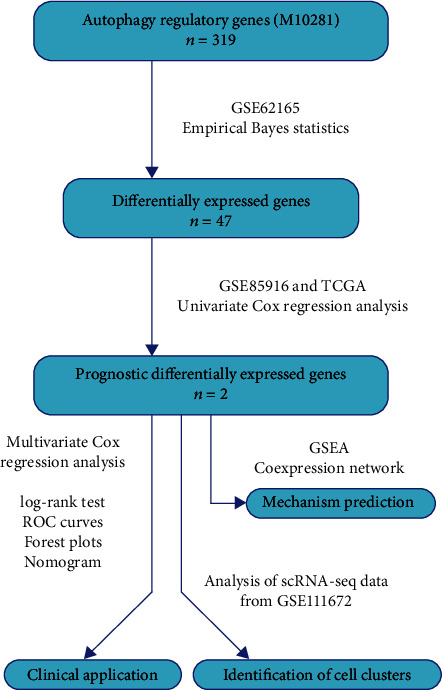
Flowchart of this study.

The differentially expressed autophagy regulatory genes and their prognostic value were analyzed by the R software. The gene expression matrixes of GSE62165 and GSE85916 were normalized using the limma package to eliminate batch effects, respectively [21]. Differentially expressed autophagy regulatory genes in PDAC compared with normal pancreatic tissues were detected in GSE62165 by empirical Bayes statistics [22]. Using the survival package, univariate Cox proportional hazards regression analysis was applied to determine the association between autophagy regulatory gene expression and overall survival (OS) of PDAC patients based on the GSE85916 group (79 samples containing complete survival data) and the TCGA group (149 samples containing complete survival data), respectively (Figure 1).

The common prognostic genes of two groups were selected to construct a risk signature using the multivariate Cox regression model, whose formula was risk score = ln[*h*(*t*, *X*)/*h*_0_(*t*)] = ∑(coe_*i*_∗exp_*i*_). Here, coe_*i*_ and exp_*i*_ were the estimated regression coefficient of the gene_*i*_ and expression of gene_*i*_, respectively. Similar methods were used to construct risk signatures based on LC3-coding genes. The risk score of every GSE85916 and TCGA sample was calculated. log-rank tests between high-risk and low-risk subgroups and receiver operating characteristic (ROC) curves using the survivalROC package were applied to evaluate and compare the predictive effectiveness of different risk signatures. Risk score distributions, scatter plots, and expression heat maps were plotted to intuitively display the risk signature's ability to distinguish samples (Figure 1).

The prognostic effect of the risk signature was also compared with that of clinicopathological factors using ROC curves in the TCGA group. Presented by forest plots, multivariate Cox regression combining risk signature and clinicopathological factors was performed to detect the independent prognostic factors. Finally, a nomogram was constructed using the rms package to represent candidate clinical application of the risk signature (Figure 1).

### 2.4. Mechanism Prediction

The functional differences of the high-risk subgroup versus the low-risk subgroup, the high-MET subgroup versus the low-MET subgroup, and the high-RIPK2 subgroup versus the low-RIPK2 subgroup were analyzed using gene set enrichment analysis (GSEA) [23]. Pearson's product-moment correlation coefficients between the expression of MET, RIPK2, and that of HADb genes were calculated to predict the mechanism of how MET and RIPK2 regulate autophagy. Only the HADb genes whose correlation coefficient with MET or RIPK2 was greater than 0.3 (*p* < 0.01) in both the GSE62165 group (118 PDAC samples) and the GSE85916 group (80 PDAC samples) were selected into the coexpression network. The coexpression network was visualized using the Cytoscape software (Figure 1).

### 2.5. Analysis of scRNA-seq Data

To identify the cells which significantly express MET and RIPK2, scRNA-seq data of two untreated PDAC patients from GSE111672 was analyzed using the Seurat package and the SingleR package in the R software. The cell numbers of patient A and patient B included in our analysis were 12000 and 7000, respectively. Similar steps were applied to these two separate datasets. Firstly, the genes which were detected in less than 70 cells and cells whose number of genes detected were less than 20 were deleted. Secondly, the count data from different batches was normalized to eliminate batch effects. Thirdly, principal component analysis (PCA) based on the remaining genes was performed to reduce variables, and then, the top 20 principal components were selected to perform *t*-distributed stochastic neighbor embedding (*t*-SNE) analysis to obtain cell clusters. Finally, the SingleR package was used to identify cell types. Scatter plots and bubble plots were plotted using the Seurat package to present the cells which expressed MET and RIPK2 (Figure 1).

### 2.6. Analysis of the Protein Expression Level of MET and RIPK2

The comparison of MET and RIPK2 protein expression in pan-cancer was gotten from the Human Protein Atlas (HPA, https://www.proteinatlas.org/).

A total of seven pairs of paraffin-embedded PDAC and corresponding adjacent tissues from patients who were clinically and pathologically diagnosed from the establishment of the Department of Hepatobiliary and Pancreatic Surgery II of the Third Xiangya Hospital in March 2019 to July 2020 were included in our immunohistochemistry (IHC) experiment. Approval from the Ethics Committee of the Third Xiangya Hospital and patients' informed consent were obtained for the use of these materials for research. Sections from paraffin-embedded samples were adhered to slides and deparaffinized with xylene and rehydrated. Then, the sections on slides were treated with citric acid antigenic retrieval buffer (pH 6.0), 3% hydrogen peroxide, and 3% bovine albumin in turn. Sections separated into two groups were incubated with anti-MET (1 : 200, Affinity, AF6128) and anti-RIPK2 (1 : 200, Affinity, DF6967) separately overnight at 4°C. The next day, they were incubated with horseradish peroxidase marked anti-rabbit secondary antibody (1 : 200, Servicebio). Then, they were treated with 3,3′-diaminobenzidine, hematoxylin in turn, dehydrated, and mounted.

## 3. Results

### 3.1. Identification of Differentially Expressed Autophagy Regulatory Genes in PDAC

**Figure 2:**
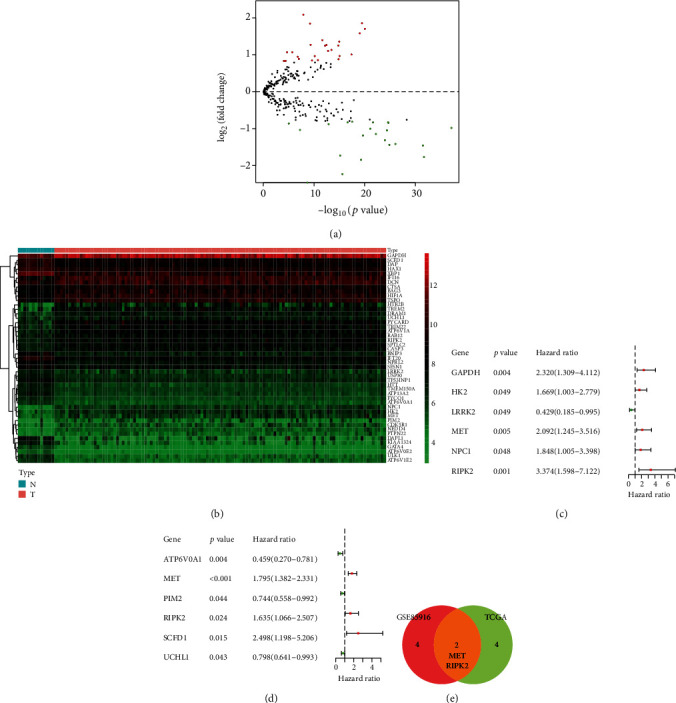
Detection of prognostic differentially expressed autophagy regulatory genes in PDAC. (a) Volcanic map of autophagy regulatory genes in GSE62165. Red dots present upregulated genes while green dots present downregulated genes in PDAC. (b) Heat map of differentially expressed autophagy regulatory genes in normal pancreatic tissue and PDAC in GSE62165. (c) Forest plot of prognostic differentially expressed autophagy regulatory genes in the GSE85916 group. (d) Forest plot of prognostic differentially expressed autophagy regulatory genes in the TCGA group. (e) Venn plot of prognostic differentially expressed autophagy regulatory genes from the GSE85916 group and the TCGA group.

We set the cut-off value of differentially expressed gene detection in GSE62165 as ∣log_2_(fold change) | >0.8 and empirical Bayes test *p* value < 0.01. As a result, 25 autophagy regulatory genes were upregulated and the 22 ones were downregulated in PDAC compared with normal pancreatic tissues (Figures 2(a) and 2(b)).

### 3.2. Identification of Prognostic Autophagy Regulatory Genes in PDAC

The univariate Cox proportional hazards regression analysis showed that 6 autophagy regulatory genes were significantly associated with OS of PDAC patients in the GSE85916 group (*p* < 0.05) (Figure 2(c)). The number of prognostic genes in the TCGA group was also 6 (*p* < 0.05) (Figure 2(d)). There were two common prognostic genes in these two groups: MET and RIPK2 (Figure 2(e)).

### 3.3. Development and Validation of the Risk Signature

**Figure 3:**
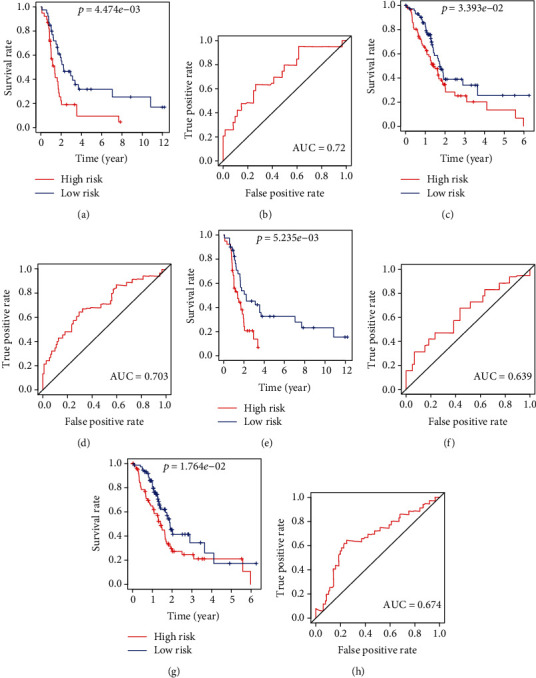
Kaplan-Meier curves and ROC curves. (a, b) Curves for risk signature constructed with MET and RIPK2 in the GSE85916 group. (c, d) Curves for risk signature constructed with MET and RIPK2 in the TCGA group. (e, f) Curves for risk signature constructed with LC3-coding genes in the GSE85916 group. (g, h) Curves for risk signature constructed with LC3-coding genes in the TCGA group.

We selected the GSE85916 group as the training group to construct a risk signature based on RNA expression of the two common prognostic genes, whose formula was risk score = (0.5265∗expression value of MET) + (0.9437∗expression value of RIPK2). The TCGA group was set as the testing group. According to the formula, we calculated the risk score of every sample of the training group and the testing group. In these two groups, respectively, we subdivided the samples into a high-risk subgroup and a low-risk subgroup based on the median risk score (7.09 and 14.61). Kaplan-Meier survival curves suggested that the prognosis of low-risk patients was significantly better than that of high-risk patients (log-rank test *p* < 0.05) (Figures 3(a) and 3(c)). In addition, we plotted the time-dependent receiver operating characteristic (ROC) curves of both groups to measure the predictive performance of the risk signature. The areas under the curve (AUC) at one year in two groups were both more than 0.7 (Figures 3(b) and 3(d)).

We constructed risk signatures based on LC3-coding genes to serve as a contrast in both groups, respectively. The formula in the GSE85916 group was risk score = (1.2104∗expression value of MAP1LC3A) + (0.8094∗expression value of MAP1LC3B) + (−0.5212∗expression value of MAP1LC3C). The formula in the TCGA group was risk score = (−0.3334∗expression value of MAP1LC3A) + (0.0950∗expression value of MAP1LC3B) + (−0.2005∗expression value of MAP1LC3B2) + (−0.1310∗expression value of MAP1LC3C). In both groups, the prognosis of low-risk patients was significantly better than that of high-risk patients (log-rank test *p* < 0.05) (Figures 3(e) and 3(g)). However, the AUC at one year were both less than 0.7 (Figures 3(f) and 3(h)), suggesting that the prognostic effect of risk signature constructed with MET and RIPK2 was better than that of risk signatures constructed with LC3-coding genes.

**Figure 4:**
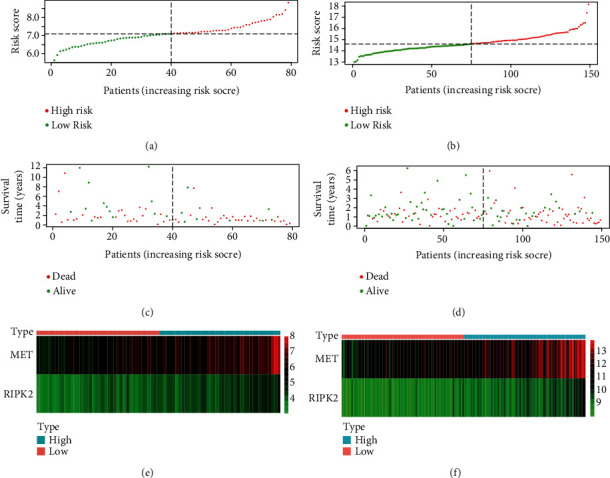
Risk scores, survival status dot plots, and heat maps of MET and RIPK2: (a, c and e) GSE85916 group; (b, d and f) TCGA group.

In both the GSE85916 group and the TCGA group, we ranked the risk scores of patients and then displayed their survival status by dot plots and the expression of MET and RIPK2 by heat maps. With the increase of risk value, the survival time of patients tended to be shorter and the proportion of deaths tend to increase, and both MET and RIPK2 tended to highly express (Figure 4).

### 3.4. Clinical Application Value of the Risk Signature Constructed with MET and RIPK2

**Figure 5:**
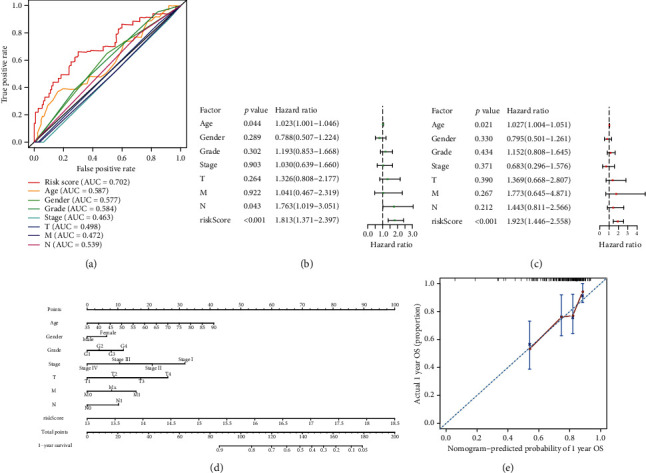
Prognostic significance and application of risk signature. (a) Multi-ROC curves of risk signature and clinicopathological factors. (b) Forest plot of univariate Cox regression analysis in the TCGA group. (c) Forest plot of multivariate Cox regression analysis in the TCGA group. (d) Nomogram to predict the one-year survival rate of PDAC patients. (e) Calibration curve of the nomogram for a one-year prediction.

In the TCGA group, we plotted ROC curves of the risk signature and clinicopathological factors to compare their prognostic effect. The AUC at one year of risk score was significantly larger than that of clinicopathological factors (Figure 5(a)). Then, we performed univariate Cox regression analysis on the risk signature and clinicopathological factors to explore their relationship with OS. As a result, risk score, age at initial pathologic diagnosis, and pathologic N were all risk factors for OS (*p* < 0.05) (Figure 5(b)). Furthermore, we performed a multivariate Cox regression analysis on the risk signature and clinicopathological factors to identify whether these factors were independently related to OS. The result showed that risk score and age at initial pathologic diagnosis were independent risk factors for PDAC prognosis (*p* < 0.05) (Figure 5(c)). Finally, we constructed a nomogram based on risk signature and clinicopathological factors to predict prognosis for PDAC patients more accurately (Figure 5(d)). Meanwhile, we plotted a calibration curve for one-year prediction to demonstrate the good predictive ability of the nomogram (Figure 5(e)). The summary statistics of the clinicopathological factors of the samples included in this step are shown in Table 1.

### 3.5. Mechanism Prediction about Risk Signature, MET, and RIPK2

**Figure 6:**
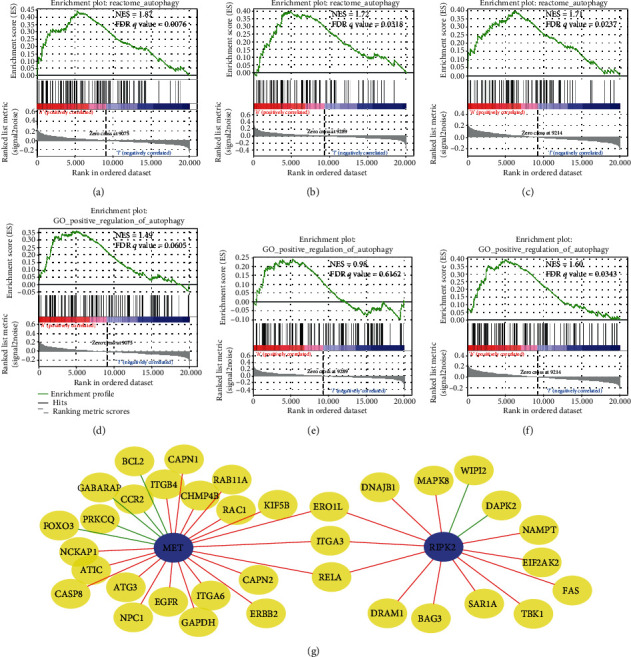
Mechanism prediction of prognostic autophagy regulation genes. (a, d) GSEA of high-risk versus low-risk. (b, e) GSEA of high-MET versus low-MET. (c, f) GSEA of high-RIPK2 versus low-RIPK2. (g) Coexpression network between MET, RIPK2, and HADb genes. Yellow nodes represent HADb genes. Red lines represent a positive correlation while green lines represent a negative correlation. NES: Normalized Enrichment Score.

We performed GSEA of 6 autophagy-related gene sets (Table 2) to demonstrate the relation between autophagy status and risk signature, MET expression, and RIPK2 expression. In the three separate analysis, REACTOME_AUTOPHAGY (M27935) were enriched in the high-risk subgroup, high-MET subgroup, and high-RIPK2 subgroup, respectively (FDR *q* value < 0.05) (Figures 6(a)–6(c)), from which we could infer that autophagy activity is upregulated in high-risk patients, high MET expression patients, and high RIPK2 expression patients. In addition, GO_POSITIVE_REGULATION_OF_AUTOPHAGY (M15852) was also enriched in the high-RIPK2 subgroup (FDR *q* value < 0.05) (Figure 6(f)), suggesting that RIPK2 probably promotes autophagy by regulating other molecules. The remaining GSEA results of other autophagy-related gene sets are presented in Supplementary Figure 1.

More specifically, we presented the probable regulatory relation between MET, RIPK2, and HADb genes by the coexpression network, predicting the mechanism of how MET and RIPK2 participate in the regulation of autophagy in PDAC (Figure 6(g)).

**Figure 7:**
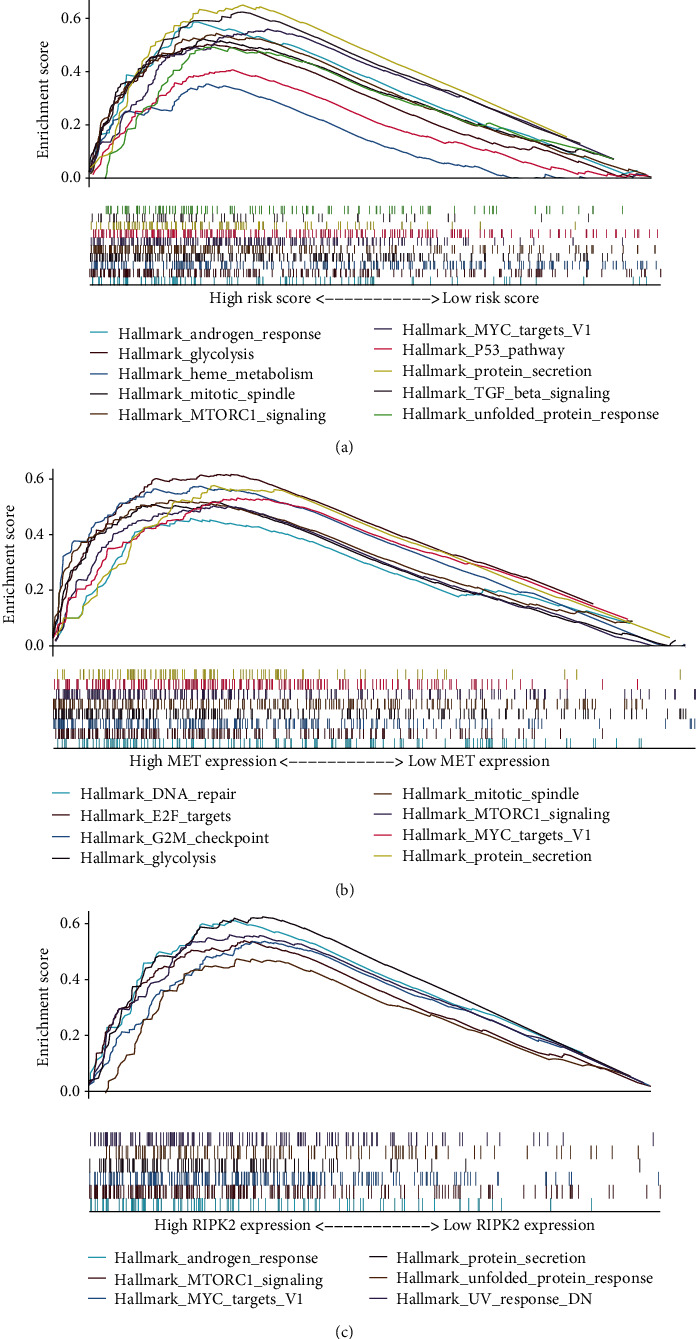
Prediction of other signaling pathways with different activities between different subgroups: (a) high-risk versus low-risk; (b) high-MET versus low-MET; (c) high-RIPK2 versus low-RIPK2.

Furthermore, we explored other signaling pathways with different activities between different subgroups. We performed GSEA concerning hallmark gene sets and KEGG gene sets. The gene sets enriched in either subgroup (FDR *q* value < 0.1) are plotted in Figure 7. HALLMARK_MTORC1_SIGNALING, HALLMARK_MYC_TARGETS_V1 and HALLMARK_PROTEIN_SECRETION were all enriched in high-risk, high-MET, and high-RIPK2 subgroups. None of KEGG gene sets was enriched.

### 3.6. Cells Which Express MET and RIPK2

**Figure 8:**
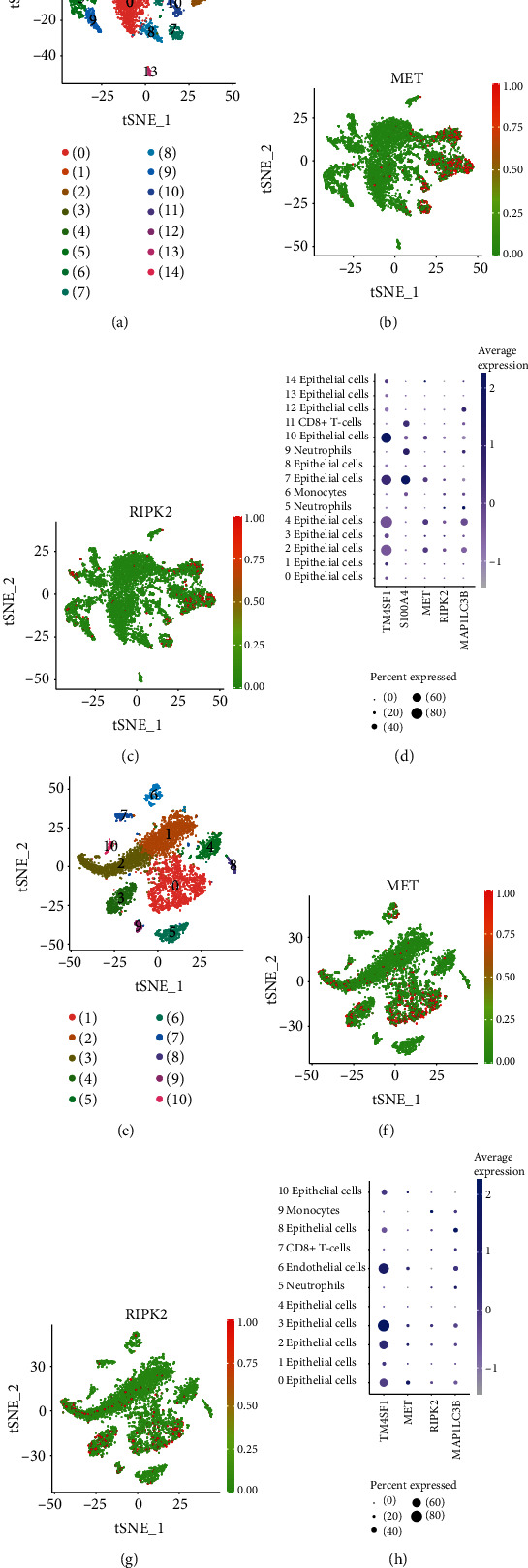
Cell clusters from the analysis of the Seurat package. Scatter plots showing normalized expression of MET and RIPK2, method of normalization: logNormalize (expression value more than 1 was set as 1). Bubble plots showing the distribution of MET, RIPK2 and MAP1LC3B expression in different cell clusters, annotated according to the analysis of the SingleR package. (a–d) patient A; (e–h) patient B.

According to our screening criteria, in the scRNA-seq data from PDAC patient A and patient B in GSE111672, the numbers of genes selected for subsequent PCA were 9992 and 9863, respectively, and the numbers of cells selected for subsequent clustering were 7448 and 6382, respectively. In the *t*-SNE analysis, we set the resolution parameters as 0.5 and 0.3 to generate 15 and 11 cell clusters, respectively, which were consistent with the analysis results of the data uploader (Figures 8(a) and 8(e)) [19]. The scatter plots suggested that MET and RIPK2 were not evenly expressed among cell clusters (Figures 8(b), 8(c), 8(f), and 8(g)). Based on the analysis results of the SingleR package (Figures 8(d) and 8(h)), we speculated that cluster 10 and cluster 7 from patient A, the epithelial cell clusters most obviously expressing TM4SF1 and S100A4, respectively, were cancer cells, while the cancer cell cluster in patient B was cluster 3, which expressed TM4SF1 most obviously [19]. As we can see from scatter plots and bubble plots, both MET and RIPK2 were expressed in cancer cells and epithelial cells. In addition, RIPK2 was also expressed in monocytes and neutrophils. In these clusters of cancer cells, monocytes, and neutrophils, MAP1LC3B was also expressed, which is transcriptionally activated in the process of autophagy [24].

### 3.7. Protein Expression Pattern of MET and RIPK2

**Figure 9:**
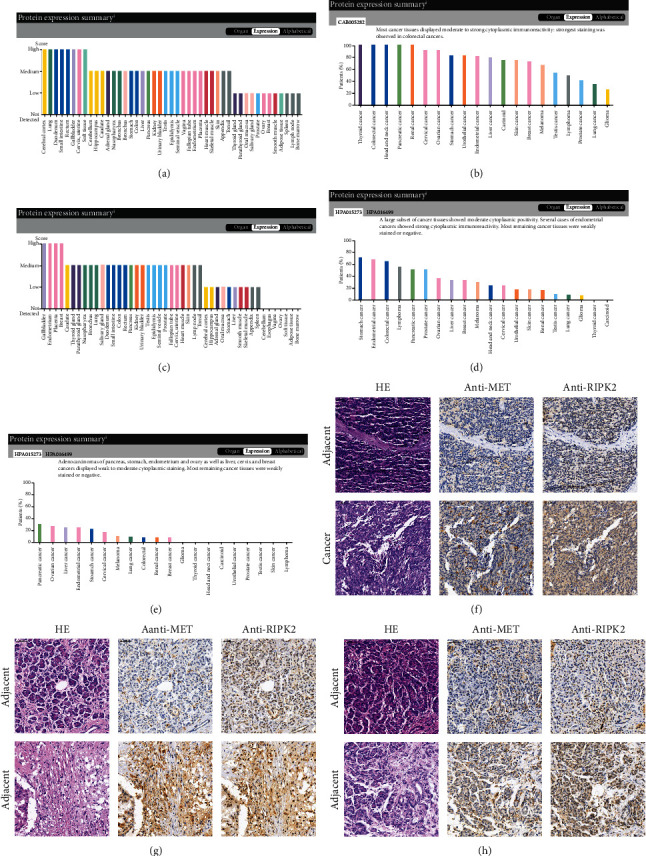
Protein expression pattern of MET and RIPK2. (a) Protein expression overview of MET in various normal organs. (b) Protein expression overview of MET in various cancers. (c) Protein expression overview of RIPK2 in various normal organs. (d, e) Protein expression overview of RIPK2 in various cancers. (f–h) Representative images of IHC staining of PDAC and adjacent pancreatic tissues (200x).

For MET and RIPK2 separately, they both express moderately in the pancreas compared with various organs (Figures 9(a) and 9(c)). However, they both highly express in pancreatic cancer compared with other cancers (Figures 9(b), 9(d), and 9(e)). As shown in the IHC images, protein expression levels of MET and RIPK2 are both elevated in PDAC compared with matching adjacent pancreatic tissue (Figures 9(f)–9(h); Supplementary Figure 2). Moreover, we can observe that MET mainly expresses in the cytoplasm while RIPK2 expresses in both the cytoplasm and the nucleus.

## 4. Discussion

In this study, a comprehensive analysis of RNA expression data from different platforms and corresponding clinical information reveals that MET and RIPK2 act as risk prognostic genes in PDAC patients and predicts the probable role of these two genes in autophagy regulation. This analysis explores the prognostic value and regulatory mechanism of autophagy regulatory genes recorded in MSigDB.

We took some measures to improve the reliability of prognosis analysis. In the GEO dataset, we chose GSE62165 and GSE85916, samples of which were selected as PDAC by strict inclusion criteria. For the samples from TCGA, we removed unqualified samples to reduce the deviation of results [17, 18]. In addition, the robust multiarray average (RMA) [25] preprocessed gene expression data of GSE85916 and the batch effects normalized mRNA expression data of TCGA from UCSC had both been log_2_(*x*) converted, being more suitable for constructing a stable generalized linear model.

The clinical application prospect of the risk signature constructed with MET and RIPK2 is worthy of expectation. A positive value of the risk signature's parameters, consistent with the heat maps ranked by risk score, and the log-rank tests between high-risk and low-risk subgroups indicated that MET, RIPK2, and risk signature are all risk factors for the prognosis of PDAC. Because the median survival time of PDAC is less than one year [3, 4], we evaluated the sensitivity and specificity of the risk signatures by ROC curves at one year. The AUC of ROC curves at one year were both greater than 0.7 in the GSE85916 group and the TCGA group, indicating good sensitivity, specificity, and stability of the risk signature constructed with MET and RIPK2. In order to provide a comparison, we constructed risk signatures based on LC3-coding genes [26, 27]. Considering that the AUC of the ROC curves at one year of the risk signatures constructed with LC3-coding genes were both less than 0.7 in two groups, we believed the clinical application of the risk signature constructed with MET and RIPK2 is more valuable in the RNA level. Moreover, the risk signature constructed with MET and RIPK2 contains only two variables, but the risk signatures constructed with LC3-coding genes contain three or four genes (GPL13667, the array platform of GSE85916, lacks a probe for MAP1LC3B2), suggesting that the former is more cost-effective. As we can see from the multi-ROC curves, compared with clinical data, the risk signature constructed with MET and RIPK2 still has obvious advantages in terms of prognostic value. The calibration curve for a one-year prediction of the nomogram showed good application potential of this risk signature combined with clinicopathological factors.

When screening differently expressed genes in GSE62165, we set the cut-off value of ∣log_2_(fold change)∣ as 0.8, rather than 1, to produce more candidate prognostic genes. Actually, the log_2_(fold change) value for MET and RIPK2 was 1.069 and 0.965, respectively. In later studies, we used IHC to further prove these expression differences. MET has been reported to be overexpressed at the protein level in PDAC, which is related to poor survival [28]. The prognostic significance of RIPK2 protein expression is worth considering to confirm. When screening the prognostic genes in GSE85916, the housekeeping gene GAPDH was calculated as a prognostic-related gene. However, in the normalized matrix of the GSE85916 group, the coefficients of variation of GAPDH, HK2, LRRK2, MET, NPC1, and RIPK2 were 0.0483, 0.0846, 0.0960, 0.1061, 0.0785, and 0.0855, respectively. The volatility of the housekeeping gene GAPDH was obviously less than other genes, which meant it is unsuitable to choose GAPDH as a prognostic indicator in clinical practice.

The mechanism prediction of MET and RIPK2 in our study provides clues for basic research. Some studies have found that MET promotes malignant phenotypes and contributes to tumor growth of PDAC [29, 30]. But it is still unclear whether MET is associated with autophagy activity in PDAC. Dephosphorylated MET was found to promote autophagy in liver cancer and gastric cancer, suggesting that a combination of kinase activity-targeted drugs and autophagy inhibitors is a potential treatment strategy [31, 32]. Part of the coexpression network about MET we constructed has been mentioned in some studies of other tumors. The expression of MET and EGFR, both of which are coding genes of receptor tyrosine kinases, is positively correlated due to the regulation of microRNA in non-small-cell lung cancer and breast cancer [33, 34]. The combination of MET inhibitors and EGFR inhibitors represents a promising therapeutic strategy [35]. In squamous cell carcinomas, EGFR is activated by MET, contributing to tumorigenesis [36]. In breast cancer, MET and ERBB2 are also coexpressed and related to therapeutic resistance [37]. RAC1 can also be activated by MET-associated complex in various cancer cells and promotes migration and invasion [38]. KIF5B gene and MET gene were reported to fuse in lung cancer, which causes elevated tumor growth [39, 40]. The role of RIPK2 in autophagy in tumors is unclear. However, RIPK2 was reported to be involved in the regulation of autophagy in intestinal bowel diseases [41–43], so it is worthy of basic experimental to research whether RIPK2 regulates autophagy in PDAC.

The analysis of scRNA-seq data reveals more details about the expression of MET and RIPK2 in PDAC. As we can see from the scatter plots, both MET and RIPK2 are expressed in cancer cells, confirming the necessity to research their role in the development of PDAC. Moreover, RIPK2 is also expressed in monocytes and neutrophils. Previous research has pointed out that autophagy not only is essential for the transformation of monocytes to macrophages [44] but also takes an important part in the major neutrophil functions [45], arousing our curiosity about the relationship between RIPK2 and autophagy in these two kinds of cells. In these cell clusters where MET or RIPK2 were expressed, MAP1LC3B, the most abundantly expressed LC3-coding genes, was also expressed, laterally confirming our hypothesis that MET and RIPK2 promote autophagy in PDAC. In addition, bubble plots showed that MET and RIPK2 are also expressed in normal epithelial cells. In the furthering research, we plan to verify our prediction by experiments in vitro and in vivo.

## 5. Conclusions

Both MET and RIPK2 are upregulated and associated with poor prognosis of PDAC. Based on the RNA expression data of these two genes, we constructed a risk signature with good prediction efficiency. In order to further research the mechanism of how MET and RIPK2 promote the progression of PDAC, we predict that the role of these two genes in cancer is to promote autophagy and took these two genes as central nodes to construct a probable regulatory network. Moreover, analysis of scRNA-seq data reveals that MET is expressed in cancer cells and epithelial cells while RIPK2 is expressed in cancer cells, epithelial cells, monocytes, and neutrophils. Our findings will be useful in the accumulation of evidence for both patient management and experiment research.

## Figures and Tables

**Table 1:** Summary statistics of clinicopathological data of PDAC patients selected into the drawing of multi-ROC curves and forest plots and construction of nomogram.

Clinicopathological factors	Variable	Count	Percentage (%)
Age at initial pathologic diagnosis	≤65 years	74	50.34
	>65 years	73	49.66
Gender	Male	68	46.26
	Female	79	53.74
Histologic grade	G1	20	13.61
	G2	83	56.46
	G3	43	29.25
	G4	1	0.68
Tumor stage	Stage I	12	8.16
	Stage II	128	87.07
	Stage III	3	2.04
	Stage IV	4	2.72
Pathologic T	T1	5	3.40
	T2	16	10.88
	T3	123	83.67
	T4	3	2.04
Pathologic M	M0	68	46.26
	Mx	75	51.02
	M1	4	2.72
Pathologic N	N0	38	25.85
	N1	109	74.15

**Table 2:** Autophagy-related gene sets we performed GSEA.

Name	Serial number	Count of genes	Organism
GO_regulation_of_autophagy	M10281	319	Homo sapiens
GO_positive_regulation_of_autophagy	M15852	114	Homo sapiens
GO_negative_regulation_of_autophagy	M12149	81	Homo sapiens
GO_selective_autophagy	M24317	47	Homo sapiens
KEGG_regulation_of_autophagy	M6382	35	Homo sapiens
Reactome_autophagy	M27935	109	Homo sapiens

## Data Availability

The RNA expression data used to support the findings of this study have been deposited in GEO and TCGA.

## Supplementary Materials

Supplementary Materials Supplementary Figure 1: remaining GSEA results with no significant statistical significance concerning the following gene sets: GO_REGULATION_OF_AUTOPHAGY (M10281), GO_NEGATIVE_REGULATION_OF_AUTOPHAGY (M12149), GO_SELECTIVE_AUTOPHAGY (M24317), and KEGG_REGULA_TION_OF_AUTOPHAGY (M6382): (a, d, g, and j) high-risk versus low-risk; (b, e, h, and k) high-MET versus low-MET; (c, f, i, and l) high-RIPK2 versus low-RIPK2. Supplementary Figure 2: remaining IHC staining of PDAC and adjacent pancreatic tissues (200x).
