# Supplementary material for: Autophagy Regulatory Genes MET and RIPK2 Play a Prognostic Role in Pancreatic Ductal Adenocarcinoma: A Bioinformatic Analysis Based on GEO and TCGA
Source: Biomed Res Int. 2020 Nov 5;2020:8537381. doi: 10.1155/2020/8537381 (PMC7665929; doi:10.1155/2020/8537381)
Supplement: Supplementary Materials — Supplementary Figure 1: remaining GSEA results with no significant statistical significance concerning the following gene sets: GO_REGULATION_OF_AUTOPHAGY (M10281), GO_NEGATIVE_REGULATION_OF_AUTOPHAGY (M12149), GO_SELECTIVE_AUTOPHAGY (M24317), and KEGG_REGULA_TION_OF_AUTOPHAGY (M6382): (a, d, g, and j) high-risk versus low-risk; (b, e, h, and k) high-MET versus low-MET; (c, f, i, and l) high-RIPK2 versus low-RIPK2. Supplementary Figure 2: remaining IHC staining of PDAC and adjacent pancreatic tissues (200x). [file 8537381.f1.zip › Supplementary Figure 1.pdf]

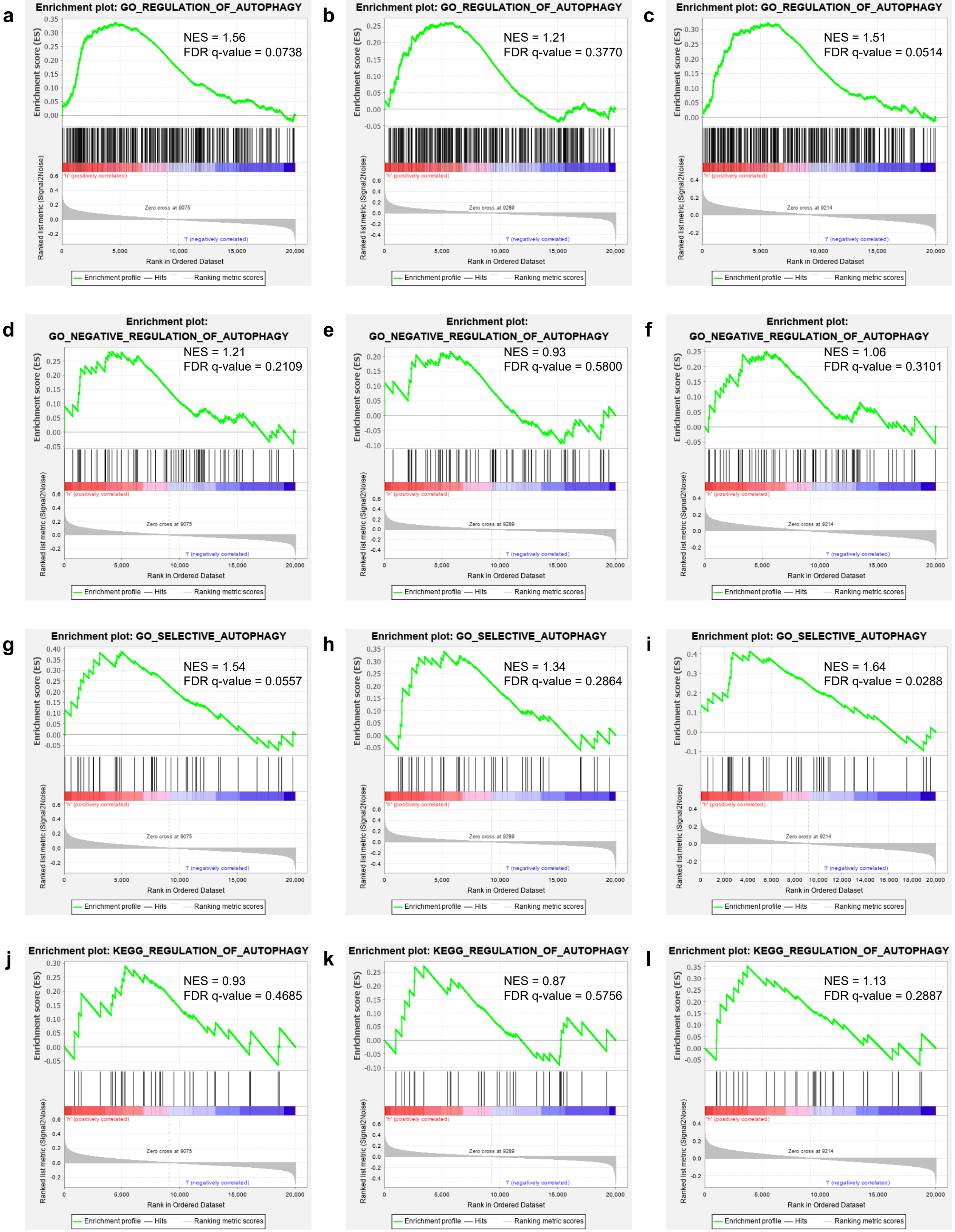

Supplementary Figure 1. Remaining GSEA results with no significant statistical significance concerning following gene sets: GO_REGULATION_OF_AUTOPHAGY (M10281), GO_NEGATIVE_REGULATION_OF_AUTOPHAGY (M12149), GO_SELECTIVE_AUTOPHAGY (M24317), KEGG_REGULATION_OF_AUTOPHAGY (M6382). (a)(d)(g)(j) high-risk versus low-risk. (b)(e)(h)(k) high-MET versus low-MET. (c)(f)(i)(l) high-RIPK2 versus low-RIPK2.
